# Comparative analysis of SDC2 and SEPT9 methylation tests in the early detection of colorectal cancer: a systematic review and meta-analysis

**DOI:** 10.3389/fmed.2024.1460233

**Published:** 2024-12-10

**Authors:** Jie Zhang, Chenhui Li, Yu An, Bing Wang, Guowei Liang

**Affiliations:** Department of Clinical Laboratory of Aerospace Center Hospital, Beijing, China

**Keywords:** SDC2, SEPT9, detection, colorectal cancer, meta-analysis

## Abstract

**Purpose:**

This meta-analysis aimed to evaluate the comparative diagnostic efficacy of Syndecan-2(SDC2) and Septin-9(SEPT9) in the early detection of colorectal cancer (CRC).

**Methods:**

We searched PubMed, Embase, Web of Science, and Cochrane Library databases to identify available publications up to October 2024. A direct head-to-head comparator analysis were performed using the random-effects model. Subgroup analyses and corresponding meta-regressions focusing on sample source, number of patients, region, study design, and methylated detection methods were conducted. Intra-group and inter-group heterogeneity were assessed by Cochrane Q and I^2^ statistics.

**Results:**

Eleven articles involving 1,913 CRC patients and 2,851 healthy people were included in the meta-analysis. The sensitivity of SDC2 was similar compared to SEPT9 for CRC patients (0.67 vs. 0.71, *p* = 0.61), SDC2 has a similar specificity in comparison to SEPT9 for CRC patients (0.90 vs. 0.91, *p* = 0.86). In subgroup analysis, stool SDC2 was similar compared to stool SEPT9 for CRC patients (sensitivity of 0.81 vs. 0.80, *p* = 0.92; specificity of 0.93 vs. 0.91, *p* = 0.73), plasma SDC2 was similar compared to plasma SEPT9 for CRC patients (sensitivity of 0.57 vs. 0.72, *p* = 0.27; specificity of 0.90 vs. 0.89, *p* = 0.89). In the subgroup analysis of clinical staging for colorectal cancer (CRC), the results indicate that there is no significant difference in sensitivity between the two markers for both early (0.7 vs. 0.67, *p* = 0.64) and advanced (0.76 vs. 0.70, *p* = 0.23) stages of CRC.

**Conclusion:**

In our head-to-head comparison meta-analysis, it was found that SDC2 and SEPT9 have similar sensitivity and specificity in the diagnosis of colorectal cancer. However, this result may be influenced by high heterogeneity and further confirmation of this finding is needed through large-scale prospective studies.

## 1 Introduction

Colorectal cancer (CRC) is a challenge in the global public health field, especially in the malignant tumors of digestive system, the incidence rate and mortality of colon cancer remain high (1). According to the GLOBOCAN database statistics released by the World Health Organization in 2020, CRC ranks the third in the global incidence rate and the second in mortality. The prognosis and diagnostic stage of cancer are closely related. The 5-year survival rate of stage I cancer patients can reach over 90%; When cancer progresses to stage IV, the 5-year survival rate significantly decreases to about 14% (2). This significant gap highlights the importance of early diagnosis and treatment. Currently, a quantitative high-risk factor questionnaire combined with fecal occult blood test (FOBT) is used as a preliminary screening method (3). However, the limitations of FOBT in identifying precancerous lesions have undoubtedly been revealed, with a sensitivity of only 33.3–57.1% (4). Colonoscopy has always been a reference standard for CRC screening, as it can visually observe the entire internal condition of the colon, but its popularity is limited (5). Therefore, non-invasive and highly sensitive CRC screening technology has become a current research hotspot and urgent need (6). Such technology should be easy to accept, reduce the burden of patients, and be able to detect CRC and precancerous lesions early and accurately, so as to help improve the early diagnosis rate and ultimately help reduce the incidence rate and mortality of CRC.

DNA methylation, as a key epigenetic mechanism, plays an important role in biological development, gene expression regulation, and diseases. Especially in cancer, abnormal DNA methylation is common and affects tumor development. Methylated DNA maintains genomic integrity and cellular function. But in cancer, abnormal methylation leads to silencing of tumor suppressor genes, promoting tumor development, such as CRC (7, 8). The Septin9 methylation detection technology can accurately identify the methylation status of the SEPT9 promoter in the blood (9), which is used for early screening of CRC. Epi proColon 2.0 is a successful commercial test kit approved by the FDA for large-scale CRC screening. However, the sensitivity of Septin9 methylation detection in early detection of CRC is limited and needs to be improved. Researchers are exploring other methylation biomarkers, such as SDC2 promoter methylation, which have high detection sensitivity and specificity in CRC and may become new detection targets (10, 11). SDC2 methylation detection has potential in early screening of CRC, but there is a lack of direct comparison studies with SEPT9 (10). The sample type may also affect the detection results. Therefore, a meta-analysis is needed to comprehensively summarize and compare the diagnostic accuracy of two methylation biomarkers, and explore the influence of sample types.

Through meta-analysis, the advantages and limitations of SDC2 methylation detection in early screening of CRC can be evaluated, and the correlation and sample type differences between the two can be explored to promote the development of early screening technology for CRC, improve prevention and control levels, and improve patient prognosis.

## 2 Methods

We follow the PRISMA-DTA guidelines for meta-analysis to ensure that the systematic evaluation and meta-analysis of diagnostic accuracy studies meet the highest quality requirements (12). PRISMA-DTA covers all aspects, and we rigorously evaluate the literature by adopting a random effects model for data integration to ensure the reliability of the conclusions.

In addition, the research protocol has been registered on PROSPERO with registration number CRD42024544612, which facilitates tracking and supervision, enhances research credibility and influence.

### 2.1 Search strategy

To comprehensively review publications on SDC2, SEPT9, and CRC up to October 2024, the research team designed a detailed literature search strategy and conducted searches on multiple internationally authoritative databases. The search scope includes medical literature databases such as PubMed and Embase, as well as knowledge service platforms such as Web of Science and Cochrane Library.

The team carefully selects keywords to ensure coverage of all outcomes from basic research to clinical applications. For detailed information, please refer to Supplementary Table S1.

To avoid missed detections, in addition to automatic search, the research reference list is also manually reviewed, and key papers are thoroughly explored to ensure that the team comprehensively and accurately grasps the latest achievements and development trends in the relationship between SDC2, SEPT9, and CRC.

### 2.2 Inclusion and exclusion criteria

In our study, the inclusion criteria were set at the following five key points:

(a) Population (P): The study subjects are individuals screened or diagnosed for colorectal cancer.
(b) Intervention (I): Evaluate the performance of SDC2 methylation test in CRC detection.
(c) Comparison (C): The study includes the SEPT9 methylation test to compare the accuracy and effectiveness of two tests in CRC screening and diagnosis.
(d) Result (O): Sensitivity and specificity were used as the main measurement indicators when evaluating the efficacy of the two experiments.
(e) Research Design (S): The included research design should include both retrospective and prospective studies.

For the exclusion criteria of the study, we set the following points:

Repeated articles, clinical guidelines, letters, case reports, comments, meta-analyses, and articles with less than 10 study cases, as well as non-English articles, will not be included in the research scope. We have also excluded articles that did not provide sufficient data to extract the sensitivity and specificity of SDC2 and SEPT9 in diagnosing CRC.

### 2.3 Quality assessment

These two researchers followed the QUADAS-2 tool standard in quality assessment, comprehensively reviewed the design, implementation, and data analysis of the included studies to ensure rigor (12).

(1) In the indicator testing phase, research the rationality of design, measurement accuracy, and the risk of bias in data collection and analysis.
(2) Reference standards, verify the application of gold standards and their compliance with clinical needs, and explore the sources of bias.
(3) In terms of traffic and timing, pay attention to recruitment, follow-up, and loss of follow-up, analyze the impact, and explore methods to reduce the risk of bias.

Ultimately, researchers accurately assessed the risk of bias at each stage, dividing it into three categories: “high risk,” “low risk,” or “unclear risk.”

### 2.4 Data extraction

Two researchers independently conducted data extraction work, selecting and obtaining the required data from a large number of studies. They delve into each research topic in detail, collect key technical details, and pay attention to the characteristics of the research design and implementation background. Features include: research country, design type; Sample source; Critical value standard; Methylation state boundary; The normal range or baseline value for assessing methylation levels. In addition, record patient level characteristics such as sample size, average age, and gender distribution. In our analysis, it is important to note that adenomas were included in the control group.

During the extraction process, researchers maintain communication and collaboration to resolve disagreements and ensure accurate and rigorous data. Collaboration avoids errors caused by personal understanding biases and ensures a solid and reliable foundation of research data.

### 2.5 Statistical analysis

In system evaluation and meta-analysis, Der Simonian and Laird methods are used to evaluate the sensitivity and specificity of the study effect size, in order to provide accurate and stable estimation results. The Freeman Tukey double sine inverse transform technique is used to quantify uncertainty and transform raw data to approach a normal distribution. In order to comprehensively understand whether there are systematic differences or inconsistencies among studies, the Jackson method was introduced to calculate confidence intervals, which is a method specifically designed for binary variable data.

For possible heterogeneity issues within different subgroups or experiments, the Cochrane Q statistic is used for analysis. Funnel plots are used to reveal potential information biases from a graphical perspective, helping researchers visually determine the existence and degree of bias by plotting the relationship between the size of each study’s effects and sample size. Finally, in order to rigorously test the existence and significance level of publication bias, Egger test was introduced. This test evaluates the impact of publication bias based on the significance of the regression intercept term. Throughout the statistical analysis process, strict standards are followed: any results involving statistical inference are only considered statistically significant when their *p*-value is less than 0.05.

In order to achieve these complex statistical analysis processes, R software version 4.1.2 was chosen as the main analysis tool, as it provides rich statistical models and plotting functions that can meet the various analysis needs mentioned above.

## 3 Results

### 3.1 Study selection

In the initial search stage of this system evaluation and meta-analysis, a strict database retrieval strategy was used to identify and obtain a total of 1,271 relevant publications. After content screening and qualification review, it was found that 541 studies had duplicate issues, 747 studies did not meet the qualification criteria, 9 out of 23 academic papers had missing or incomplete data, 2 non-English articles were excluded, and 1 article did not provide positive rate. Finally, 11 high-quality research articles were selected for inclusion in this meta-analysis (11, 13–21). These studies are all from authoritative publishers and have high-quality research designs and data reporting standards.

**Figure 1:**
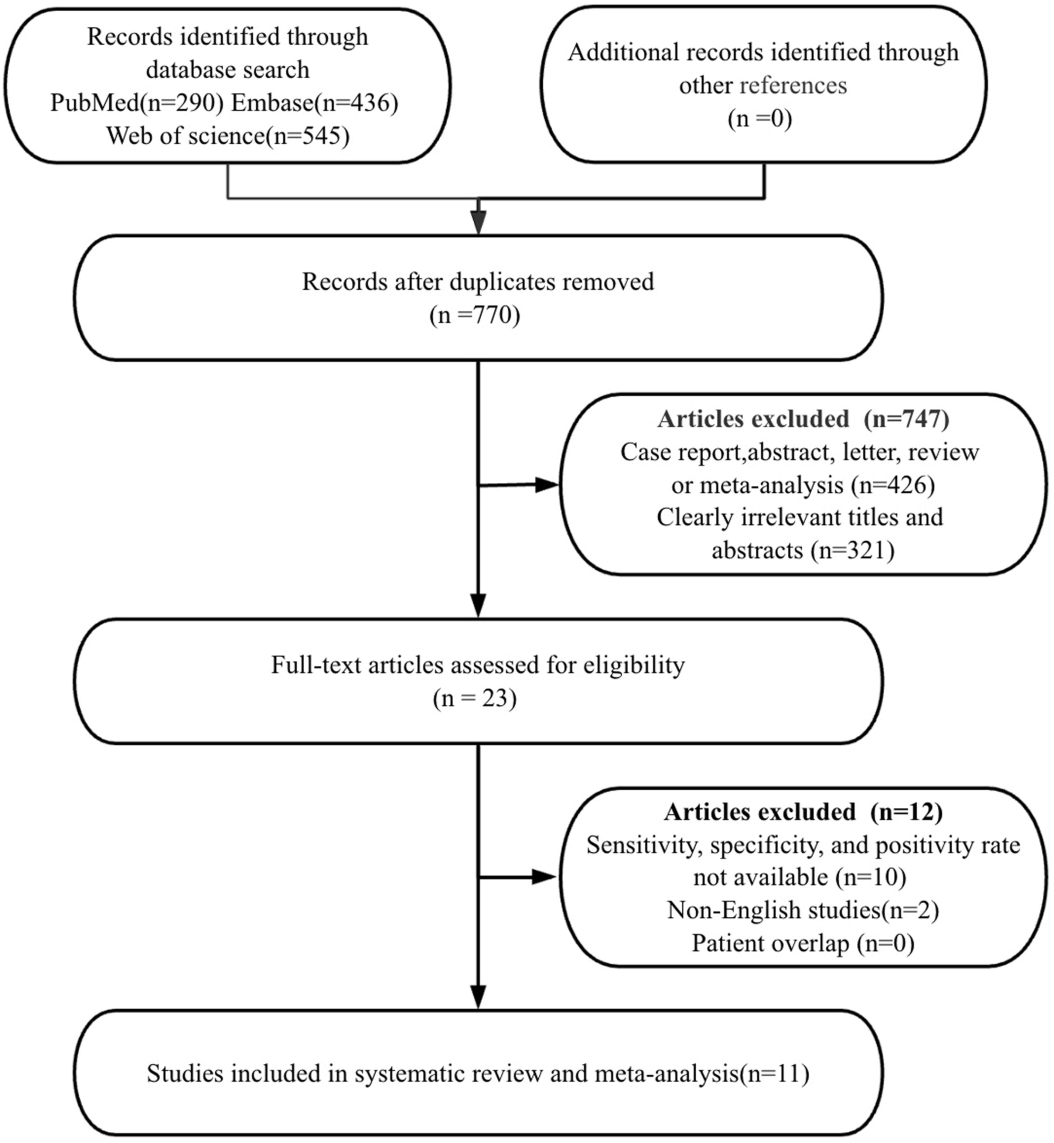
PRISMA flow diagram illustrating the study selection process.

According to the PRISMA flowchart, the detailed steps and results of the entire article selection process were depicted in Figure 1.

### 3.2 Study description and quality assessment

The 11 eligible studies encompassed a total of 1913 CRC patients and 2,851 healthy individuals from two countries: China and France. Out of these studies, 2 had a retrospective design, while 9 were prospective. In terms of sample sources, 6 studies utilized blood samples, and 3 studies used stool samples. Notably, the study by Zhan et al. (19) and Zou et al. (22) compared stool-derived SDC2 with blood-derived SEPT9. Ten studies employed Bisulfite conversion-qMSP (quantitative methylation-specific PCR) as the method for detecting methylation, while 1 study used Enzyme Digestion-qMSP. Table 1 provides a summary of the study and technique characteristics of the included studies.

**Table 1:** Study and patient characteristics of the included studies.

Author	Year	Country	Study design	Sample source	Methylated detection methods	Cut-off (SDC2/Septin9)	CRC			Control			Reference standard
							No. of patients	Mean age	Male/Female	No. of patients	Mean age	Male/Female	
Zou et al. (22)	2024	China	Prospective	Stool/ Blood	Bisulfite conversion-qMSP	38/35	116	60.4	71/45	75	59.3	39/36	Colonoscopy
Zhan et al. (19)	2024	China	Prospective	Stool/ Blood	Bisulfite conversion-qMSP	48/42	445/340	NA	295/150	557	NA	NA	Colonoscopy
Li et al. (16)	2023	China	Prospective	Blood	Bisulfite conversion-qMSP	40/45	75	57	54/21	211	51.5	128/123	Colonoscopy
Dai et al. (15)	2022	China	Retro	Stool	Bisulfite conversion-qMSP	40/38	102	61	56/46	186	51	90/96	Colonoscopy
Xu et al. (11)	2021	China	Prospective	Blood	Bisulfite conversion-qMSP	44.5/41.9	104	64.7	65/39	190	59.6	127/63	Colonoscopy
Liu et al. (17)	2021	China	Prospective	Stool	Enzyme Digestion-qMSP	42	180	NA	NA	962	NA	NA	Colonoscopy
Chen et al. (14)	2021	China	Prospective	Blood	Bisulfite conversion-qMSP	50/45	91	62.7	55/36	122	51.5	79/43	Colonoscopy
Zhao et al. (21)	2020	China	Prospective	Stool	Bisulfite conversion-qMSP	50/45	39	59	21/18	65	48.7	34/31	Colonoscopy
Zhao et al. (20)	2019	China	Prospective	Blood	Bisulfite conversion-qMSP	50/45	117	61.8	64/53	267	44.2	150/117	Colonoscopy
Chen et al. (13)	2019	China	Prospective	Blood	Bisulfite conversion-qMSP	50	111	61	75/36	114	33.2	NA	Colonoscopy
Rasmussen et al. (18)	2017	France	Retro	Blood	Bisulfite conversion-qMSP	NA	193	67.5	119/74	102	64.7	55/47	Colonoscopy

NA, not available; q-MSP, quantitative methylation-specific polymerase chain reaction.

**Figure 2:**
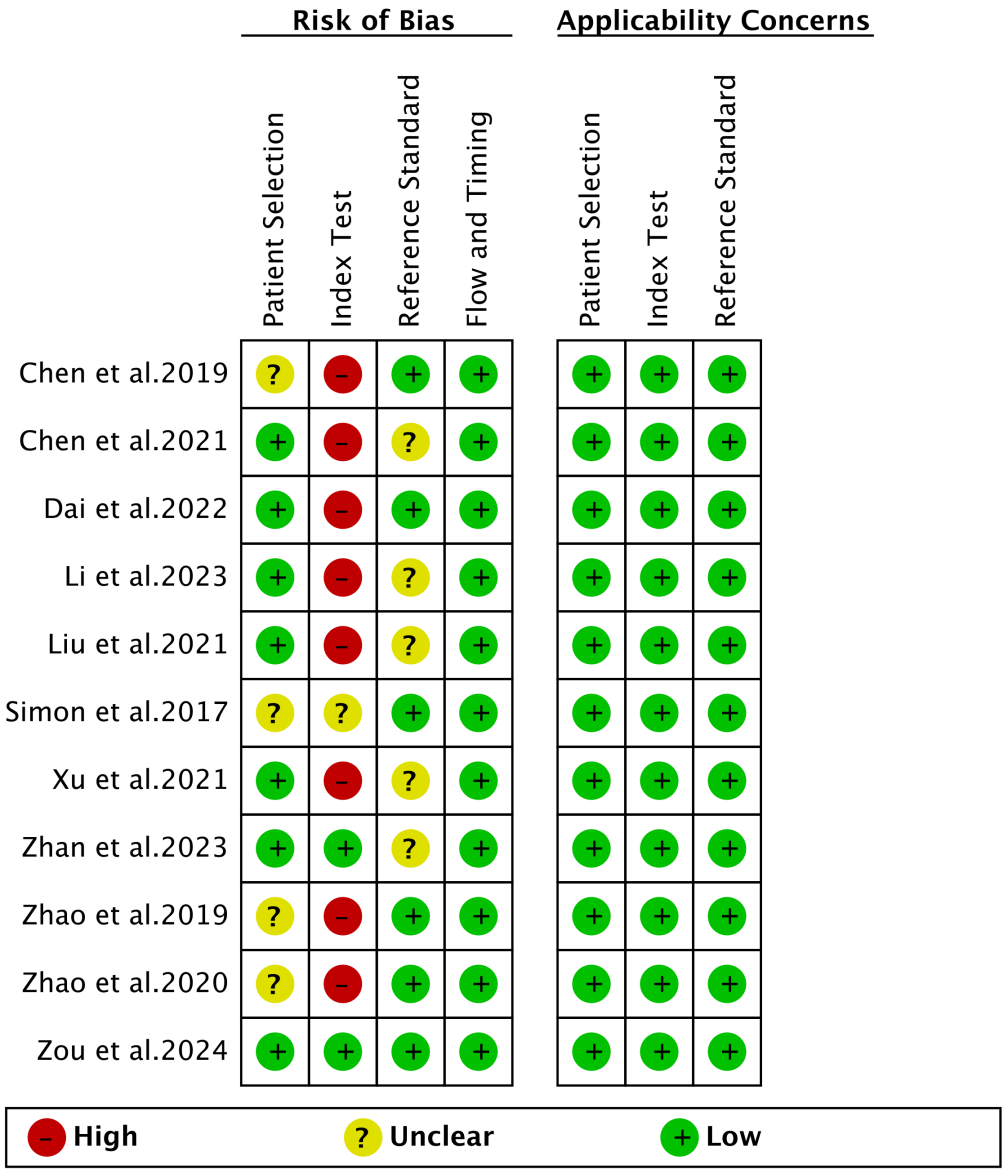
Risk of bias and applicability concerns of the included studies using the quality assessment of diagnostic performance studies QUADAS-2 tool.

The risk of bias for each study, assessed using the QUADAS-2 tool, is illustrated in Figure 2. In the assessment of patient selection risk of bias, four studies were rated as “unclear” due to the lack of information on whether they included consecutive patients. For the index test, one study was rated as “unclear” because it did not provide information on whether the applied cut-off values were predetermined. Eight studies were rated as “high risk” because the cut-off values were based on the Youden index determined by the samples. Regarding the reference standard, five studies were rated as “unclear” as the final diagnosis was not independently determined by two or more physicians. The flow and timing standard were rated as “low risk” in all ten studies. Based on the overall quality assessment, there were no major concerns regarding the quality of the included studies.

### 3.3 Comparing the sensitivity of SDC2 and SEPT9 for detecting CRC

**Figure 3:**
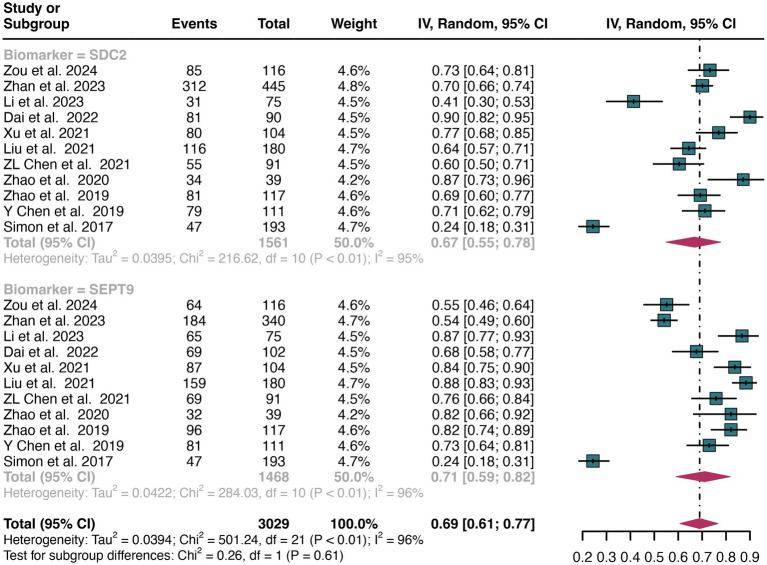
Forest plot showing the pooled sensitivities of SDC2 and SEPT9 in CRC patients on a patient-based analysis. The plot displays individual study estimates (squares) with corresponding 95% confidence intervals (horizontal lines) and the pooled sensitivity estimate (diamond) for both modalities. The size of the squares represents the relative weight of each study in the meta-analysis.

A total of 11 studies were included in the analysis. The combined sensitivity of SDC2 in detecting CRC was found to be 0.67 (95% CI: 0.55–0.78), while SEPT9 exhibited a comparable sensitivity of 0.71 (95% CI: 0.59–0.82) (Figure 3). There was no statistically significant difference in sensitivity between SDC2 and SEPT9 (*p* = 0.61) (Figure 3).

The overall sensitivity of SDC2 and SEPT9 showed I^2^ values of 95 and 96%, respectively. For SDC2, meta-regression analysis revealed that the region (Asia vs. Non-Asia, *p* < 0.01) could be a potential source of heterogeneity (Table 2). Similarly, for SEPT9, meta-regression analysis indicated that both the region (Asia vs. Non-Asia, *p* < 0.01) and the study design (retrospective vs. prospective, *p* = 0.03) might contribute to heterogeneity (Table 3). However, leave-one-out sensitivity analysis did not identify any specific source of heterogeneity (Supplementary Figures S1, S2).

**Table 2:** Subgroup analysis and meta-regression analysis for Syndecan-2.

Covariate	Studies, *n*	Sensitivity (95%CI)	*P*-value	Specificity(95%CI)	*p*-value
No. of patients			0.74		0.02
≤100	3	0.64(0.36–0.87)		0.84(0.72–0.93)	
>100	8	0.68(0.54–0.80)		0.93(0.90–0.96)	
Region			<0.01		0.64
Asia	10	0.71 (0.62–0.79)		0.91(0.86–0.95)	
Non- Asia	1	0.24 (0.18–0.31)		0.94(0.88–0.98)	
Study design			0.50		0.88
Retrospective	2	0.59(0.02–1.00)		0.91(0.87–0.95)	
Prospective	9	0.68(0.61–0.76)		0.91(0.86–0.95)	
Methylated detection methods			0.90		0.38
Bisulfite conversion-qMSP	10	0.67(0.54–0.79)		0.91(0.86–0.94)	
Enzyme Digestion-qMSP	1	0.64(0.57–0.71)		0.95(0.94–0.97)	
Sample source			0.06		0.72
Blood	6	0.57(0.40–0.74)		0.90(0.83–0.96)	
Stool	5	0.77(0.66–0.86)		0.92(0.87–0.96)	

q-MSP, quantitative methylation-specific polymerase chain reaction.

**Table 3:** Subgroup analysis and meta-regression analysis for Septin 9.

Covariate	Studies, *n*	Sensitivity (95%CI)	*P*-value	Specificity(95%CI)	*P*-value
No. of patients			0.30		0.09
≤100	3	0.81(0.74–0.88)		0.80(0.40–1.00)	
>100	7	0.67(0.51–0.81)		0.93(0.90–0.96)	
Region			<0.01		0.67
Asia	9	0.75(0.67–0.83)		0.90(0.80–0.97)	
Non-Asia	1	0.24(0.18–0.31)		0.95(0.89–0.98)	
Study design			0.03		0.47
Retrospective	2	0.45(0.09–0.86)		0.96(0.93–0.98)	
Prospective	8	0.76(0.67–0.84)		0.89(0.78–0.97)	
Methylated detection methods			0.30		0.75
Bisulfite conversion-qMSP	9	0.69(0.56–0.80)		0.91(0.82–0.97)	
Enzyme Digestion-qMSP	1	0.88(0.83–0.93)		0.85(0.83–0.88)	
Sample source			0.37		0.88
Blood	6	0.68(0.52–0.81)		0.90(0.78–0.98)	
Stool	3	0.80(0.66–0.91)		0.91(0.84–0.97)	

*q-MSP quantitative methylation-specific polymerase chain reaction.*

### 3.4 Comparing the specificity of SDC2and SEPT9 for detecting CRC

**Figure 4:**
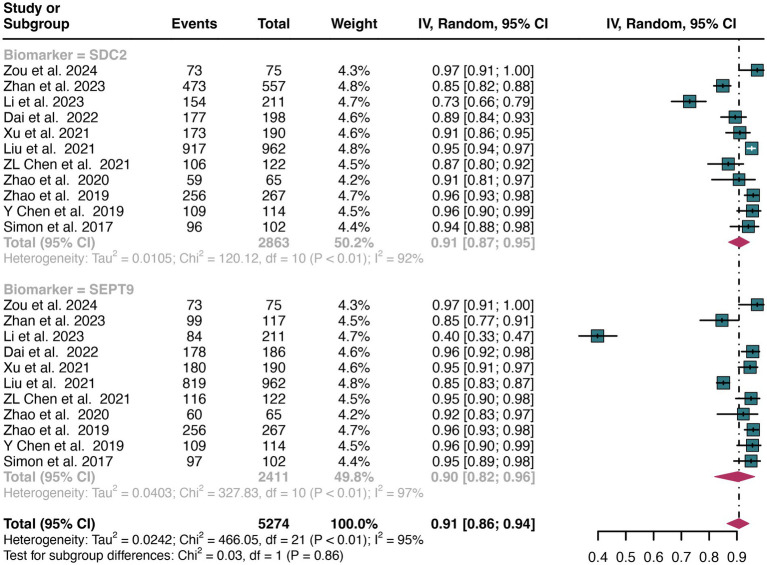
Forest plot showing the pooled specificities of SDC2 and SEPT9 in CRC patients on a patient-based analysis. The plot displays individual study estimates (squares) with corresponding 95% confidence intervals (horizontal lines) and the pooled sensitivity estimate (diamond) for both modalities. The size of the squares represents the relative weight of each study in the meta-analysis.

A total of 11 studies were included in the analysis. The pooled specificity of SDC2 in detecting CRC was 0.91 (95% CI: 0.87–0.95), whereas SEPT9 had the same specificity of 0.90 (95% CI: 0.82–0.96) (Figure 4). There was no significant difference in specificity between SDC2 and SEPT9 (*p* = 0.86) (Figure 4).

The overall specificity of SDC2 and SEPT9 exhibited I^2^ values of 92 and 97%, respectively. For SDC2, meta-regression analysis found that the number of patients (<100 vs. >100, *p* = 0.02) was a possible source of heterogeneity (Table 2). Leave-one-out sensitivity analysis revealed no source of heterogeneity (Supplementary Figures S3, S4).

### 3.5 Subgroup analysis based on sample source: comparing the sensitivity of SDC2 and SEPT9 in detecting CRC

**Figure 5:**
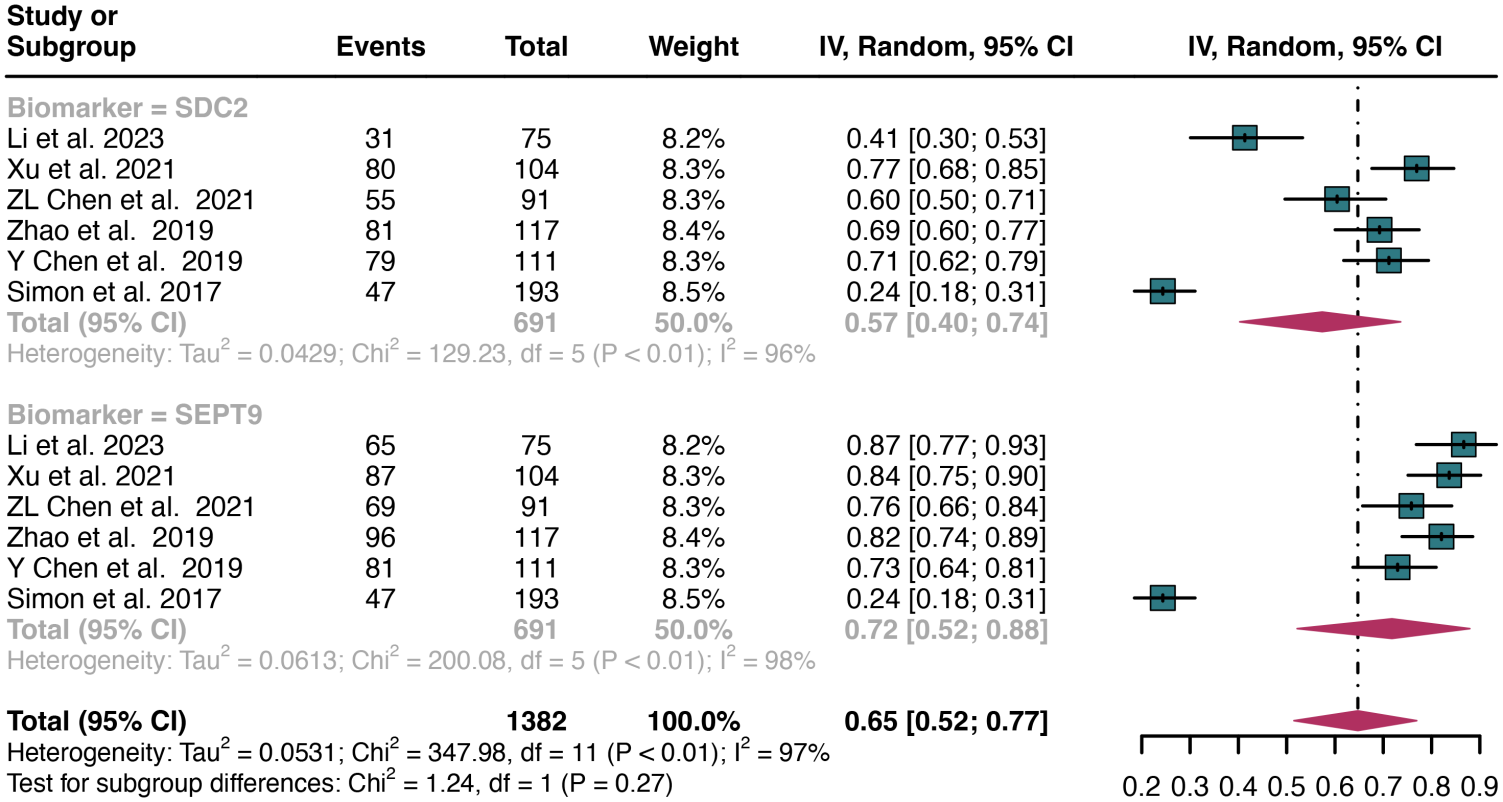
Forest plot showing the head-to-head comparison of sensitivities for SDC2 and SEPT9 in plasma in CRC patients. The plot displays individual study estimates (squares) with corresponding 95% confidence intervals (horizontal lines) and the pooled sensitivity estimate (diamond) for both modalities. The size of the squares represents the relative weight of each study in the meta-analysis.

For plasma sample, a total of 6 studies were included in the analysis. The pooled sensitivity of SDC2 in detecting CRC was 0.57 (95% CI: 0.40–0.74), whereas SEPT9 had a similar sensitivity of 0.72 (95% CI: 0.52–0.88) (Figure 5). There was no significant difference in sensitivity between SDC2 and SEPT9 (*p* = 0.27) (Figure 5).

**Figure 6:**
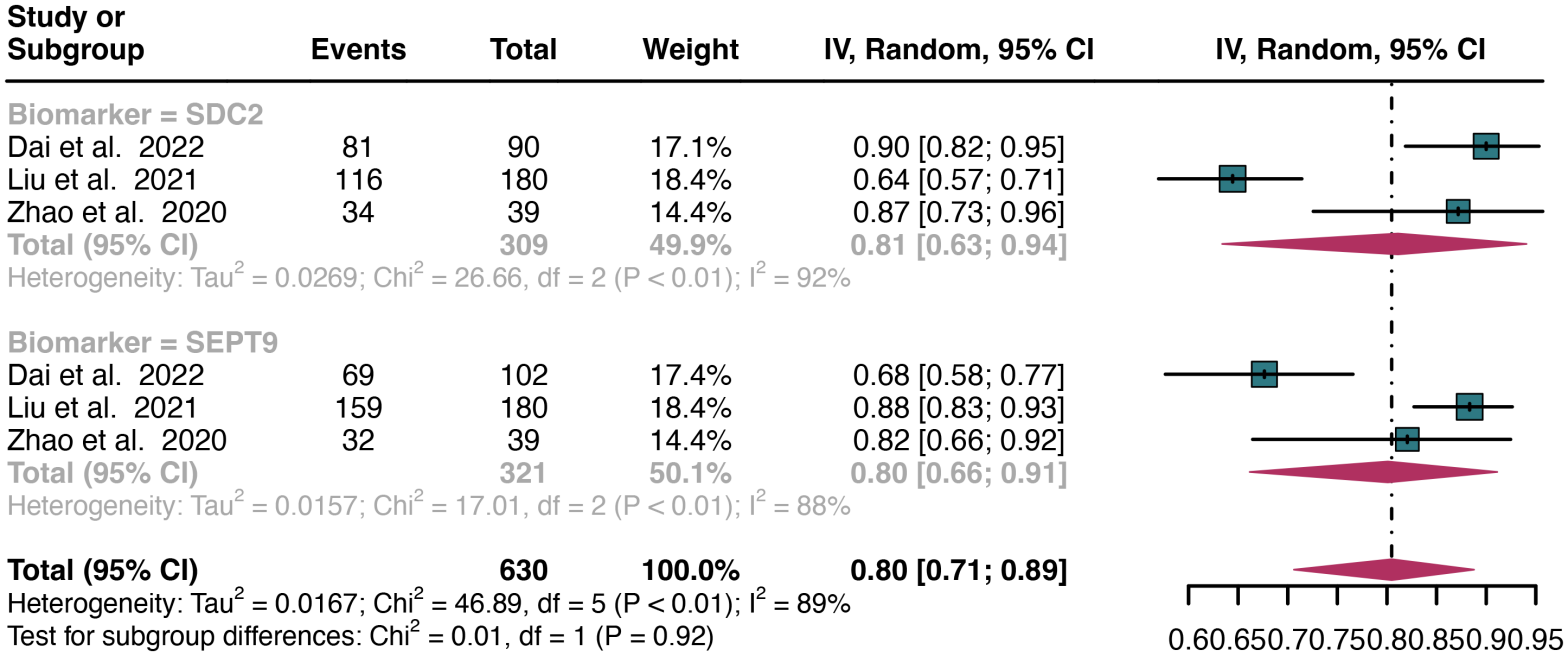
Forest plot showing the head-to-head comparison of sensitivities for SDC2 and SEPT9 in stool in CRC patients. The plot displays individual study estimates (squares) with corresponding 95% confidence intervals (horizontal lines) and the pooled sensitivity estimate (diamond) for both modalities. The size of the squares represents the relative weight of each study in the meta-analysis.

For stool sample, a total of 3 studies were included in the analysis. The pooled sensitivity of SDC2 in detecting CRC was 0.81 (95% CI: 0.63–0.94), whereas SEPT9 had a similar sensitivity of 0.80 (95% CI: 0.66–0.91) (Figure 6). There was no significant difference in sensitivity between SDC2 and SEPT9 (*p* = 0.92) (Figure 6).

### 3.6 Subgroup analysis based on sample source: comparing the specificity of SDC2 and SEPT9 in detecting CRC

**Figure 7:**
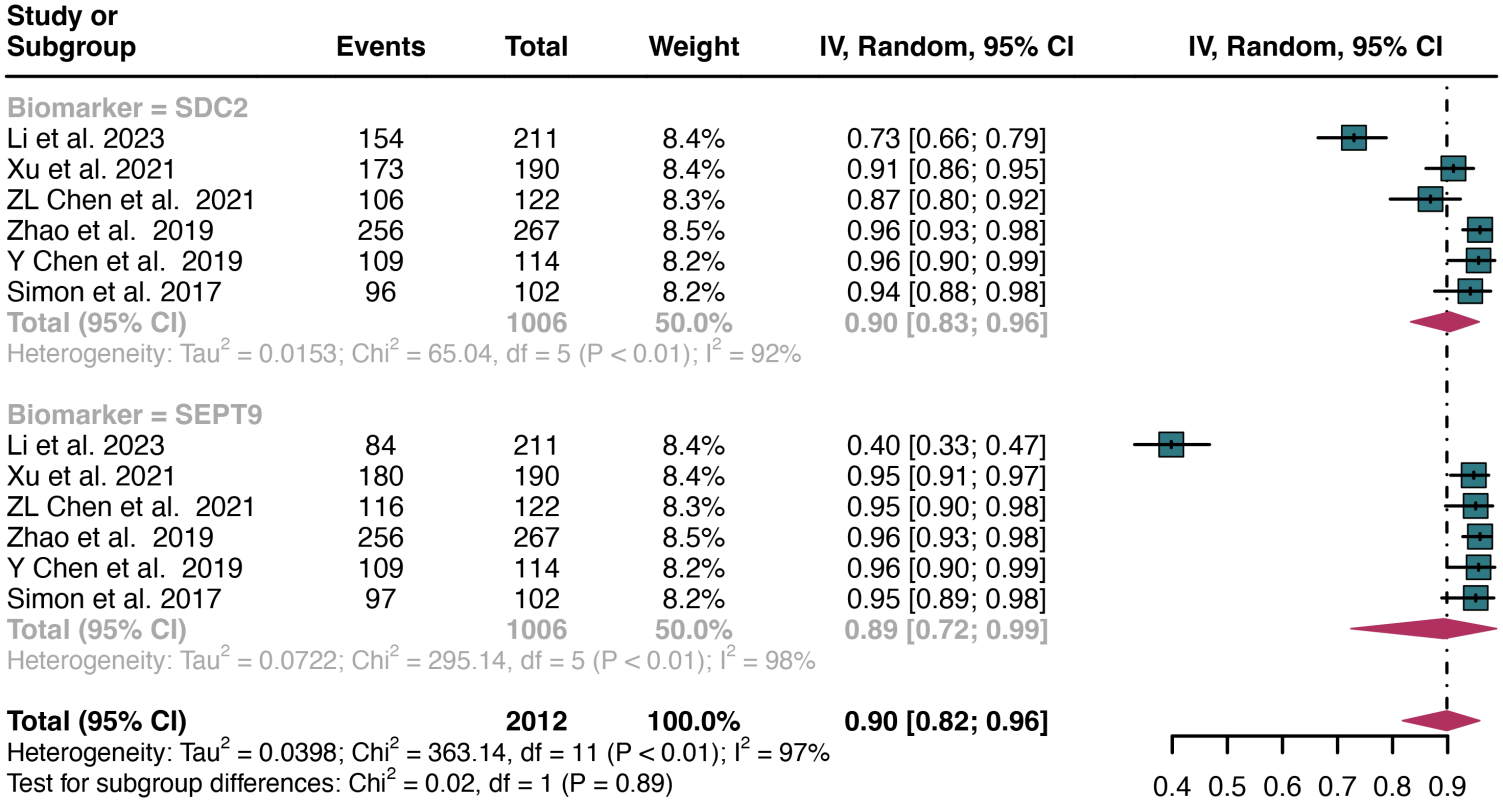
Forest plot showing the head-to-head comparison of specificities for SDC2 and SEPT9 in plasma in CRC patients. The plot displays individual study estimates (squares) with corresponding 95% confidence intervals (horizontal lines) and the pooled sensitivity estimate (diamond) for both modalities. The size of the squares represents the relative weight of each study in the meta-analysis.

For plasma sample, the pooled specificity of SDC2 in detecting CRC was 0.90 (95% CI: 0.83–0.96), whereas SEPT9 had a similar specificity of 0.89 (95% CI: 0.72–0.99) (Figure 7). There was no significant difference in specificity between SDC2 and SEPT9 (*p* = 0.89) (Figure 7).

**Figure 8:**
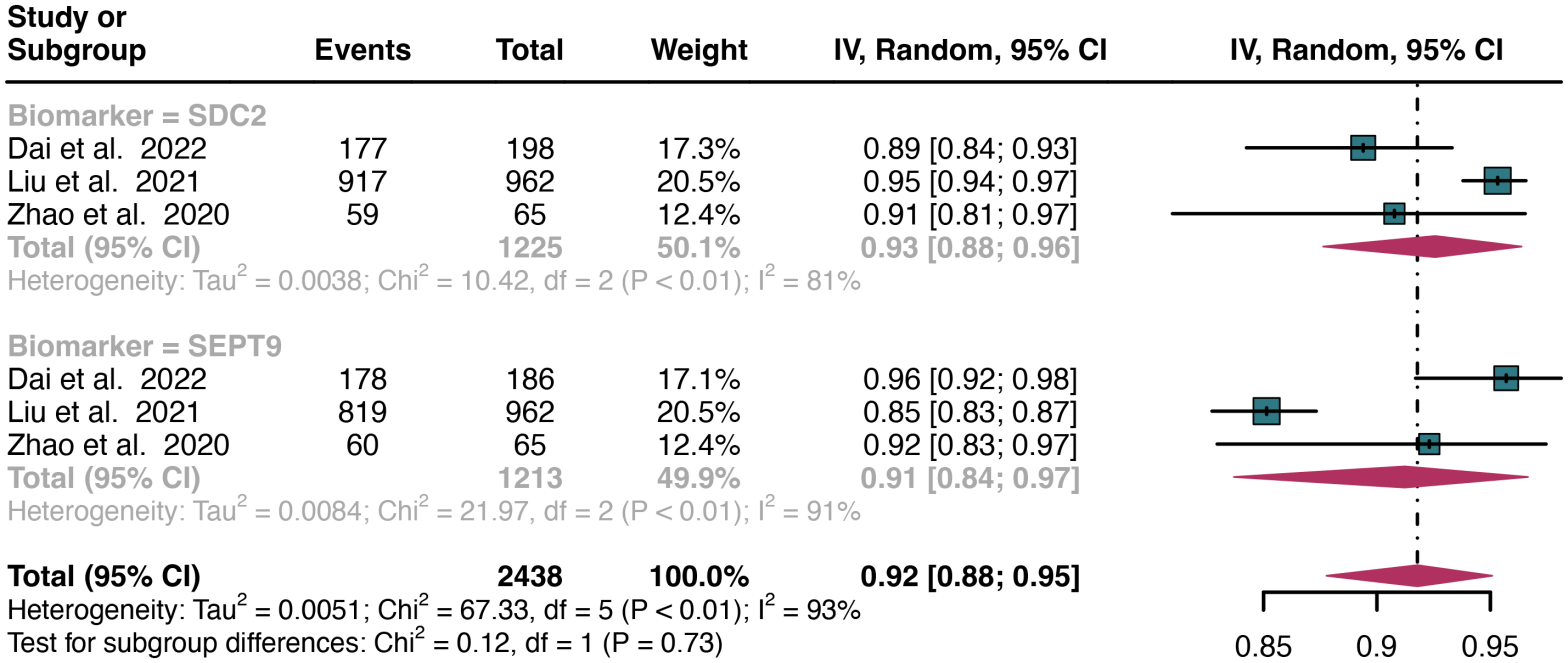
Forest plot showing the head-to-head comparison of specificities for SDC2 and SEPT9 in stool in CRC patients. The plot displays individual study estimates (squares) with corresponding 95% confidence intervals (horizontal lines) and the pooled sensitivity estimate (diamond) for both modalities. The size of the squares represents the relative weight of each study in the meta-analysis.

For stool sample, the pooled specificity of SDC2 in detecting CRC was 0.93 (95% CI: 0.88–0.96), whereas SEPT9 had a similar specificity of 0.91 (95% CI: 0.84–0.97) (Figure 8). There was no significant difference in specificity between SDC2 and SEPT9 (*p* = 0.73) (Figure 8).

### 3.7 Subgroup analysis based on CRC stage: comparing the sensitivity of SDC2 and SEPT9 in detecting CRC

**Figure 9:**
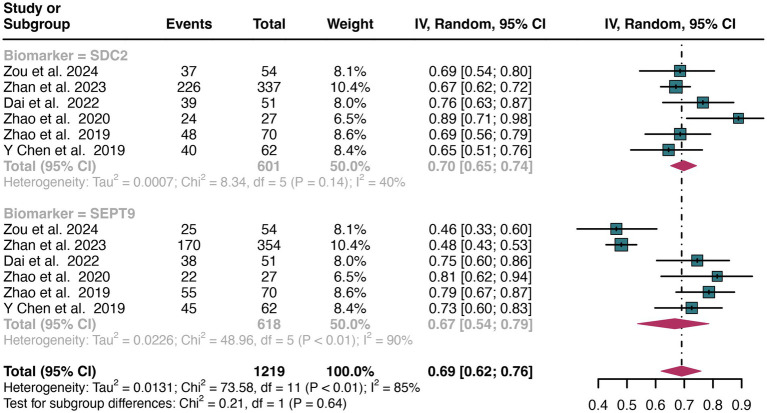
Forest plot showing the head-to-head comparison of sensitivities for SDC2 and SEPT9 in stool in early stage of CRC patients. The plot displays individual study estimates (squares) with corresponding 95% confidence intervals (horizontal lines) and the pooled sensitivity estimate (diamond) for both modalities. The size of the squares represents the relative weight of each study in the meta-analysis.

For early stage, a total of 6 studies were included in the analysis. The pooled sensitivity of SDC2 in detecting early stage of CRC was 0.70 (95% CI: 0.65–0.74), whereas SEPT9 had a similar sensitivity of 0.67 (95% CI: 0.54–0.79) (Figure 9). There was no significant difference in sensitivity between SDC2 and SEPT9 (*p* = 0.64) (Figure 9).

**Figure 10:**
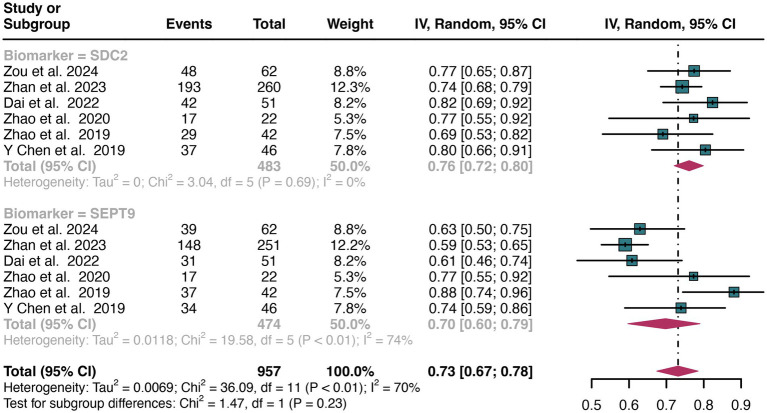
Forest plot showing the head-to-head comparison of sensitivities for SDC2 and SEPT9 in stool in late stage of CRC patients. The plot displays individual study estimates (squares) with corresponding 95% confidence intervals (horizontal lines) and the pooled sensitivity estimate (diamond) for both modalities. The size of the squares represents the relative weight of each study in the meta-analysis.

For late stage, the same 6 studies were included in the analysis. The pooled sensitivity of SDC2 in detecting late stage of CRC was 0.76 (95% CI: 0.72–0.80), whereas SEPT9 had a similar sensitivity of 0.70 (95% CI: 0.60–0.79) (Figure 10). There was no significant difference in sensitivity between SDC2 and SEPT9 (*p* = 0.23) (Figure 10).

### 3.8 SROC curve for SDC2 and SEPT9

**Figure 11:**
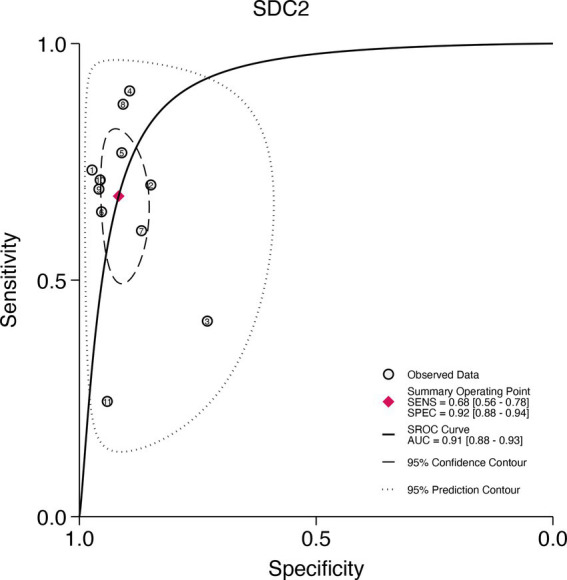
Summary receiver operating characteristic (SROC) curve analysis of SDC2 methylation tests. The circles represent the observed data, the squares indicate the summary operating point, the solid line denotes the SROC curve, the long-dashed line represents the 95% confidence contour, and the short-dashed line indicates the 95% prediction contour.

**Figure 12:**
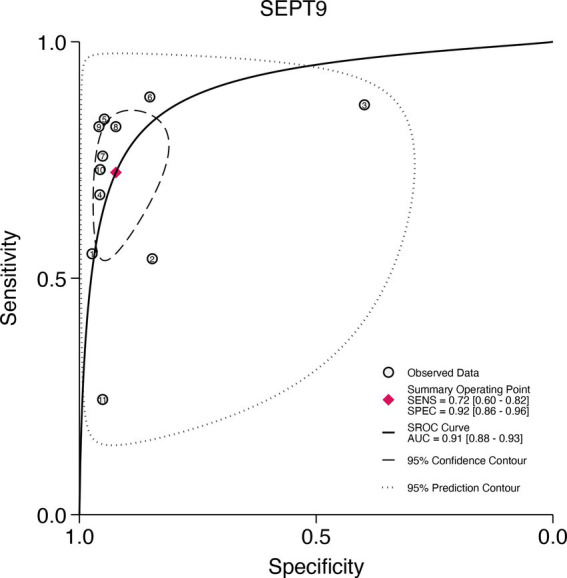
Summary receiver operating characteristic (SROC) curve analysis of SEPT9 methylation tests. The circles represent the observed data, the squares indicate the summary operating point, the solid line denotes the SROC curve, the long-dashed line represents the 95% confidence contour, and the short-dashed line indicates the 95% prediction contour.

This study plotted the summary SROC curves for SDC2 and SEPT9. The optimal cutoff point for SDC2 was a sensitivity of 0.68 (0.56–0.78) and a specificity of 0.92 (0.88–0.94), with an area under the SROC curve (AUC) of 0.91 (0.88–0.93) (Figure 11). For SEPT9, the optimal cutoff point was a sensitivity of 0.72 (0.60–0.82) and a specificity of 0.92 (0.86–0.96), with an AUC of 0.91 (0.88–0.93) (Figure 12).

### 3.9 Publication bias

Funnel plot asymmetry test showed that no significant publication bias was observed for all of outcome (Egger’s test: all *p* > 0.05) (Supplementary Figures S5–S8).

## 4 Discussion

In April 2016, the FDA approved a blood test designed to detect circulating methylated SEPT9 DNA (EpiproColon;Epigenomics) (23). This approval was based on a single test characteristic study that met the inclusion criteria for a systematic evidence review (24). The study found that the SEPT9 DNA test demonstrated low sensitivity, detecting colorectal cancer in only 48% of cases (24). Consequently, there has been a growing interest in identifying a new, more sensitive methylation site to enhance the efficacy of colorectal cancer screening. In recent years, SDC2 has emerged as a promising methylation site, with various studies indicating that SDC2 possesses good diagnostic performance for colorectal cancer (10). However, there remains a lack of systematic comparison between SDC2 and SEPT9 to determine which marker offers superior diagnostic accuracy. While individual studies have shown potential, a comprehensive analysis is needed to definitively ascertain the diagnostic efficacy of SDC2 relative to SEPT9. Therefore, this meta-analysis aims to fill this gap by systematically comparing the diagnostic performance of these two methylation markers.

The results of this study indicate that the sensitivity of SDC2 is comparable to that of SEPT9 for CRC patients, with sensitivity values of 0.67 for SDC2 and 0.71 for SEPT9 (*p* = 0.61). Additionally, SDC2 exhibited similar specificity to SEPT9 for CRC patients, with specificity values of 0.91 for SDC2 and 0.90 for SEPT9 (*p* = 0.86). In subgroup analyses, stool SDC2 showed similar sensitivity and specificity to stool SEPT9 for CRC patients, with sensitivities of 0.81 vs. 0.80 (*p* = 0.92) and specificities of 0.93 vs. 0.91 (*p* = 0.73). Plasma SDC2 also demonstrated comparable results to plasma SEPT9, with sensitivities of 0.57 vs. 0.72 (*p* = 0.27) and specificities of 0.90 vs. 0.89 (*p* = 0.89). Additionally, the diagnostic sensitivity of SDC2 and SEPT9 is similar for both early and advanced stages of colorectal cancer. The comparable performance of SDC2 and SEPT9 can be attributed to their similar mechanisms of detecting methylated DNA markers associated with CRC, suggesting that SDC2 is as effective as SEPT9 in identifying CRC patients. Septin9 is a group of scaffold proteins that provide structural support during cell division (25). High methylation of its promoter region, accompanied by transcriptional damage, leads to loss of anticancer activity and promotes the malignant progression of colorectal lesions (25). The CpG island 3 in the promoter region of the V2 transcript of the SEPT9 gene is highly methylated (26). During the development of colorectal cancer, the DNA of this gene is released from necrotic and apoptotic cancer cells into the peripheral circulation (26). The risk of colorectal cancer can be determined by detecting the degree of DNA methylation in specific promoter regions of the SEPT9 gene in peripheral blood (27). SDC2 is a transmembrane glycoprotein that participates in cell proliferation, migration, and cell matrix interactions through extracellular matrix protein receptors (28). Methylation of SDC2 leads to transcriptional silencing, disrupted cell growth and differentiation, and massive proliferation of tumor cells, exhibiting strong invasive activity and metastatic characteristics (28). Therefore, SDC2 methylation can be detected in detached cancer cells. Fallen cancer cells can appear in both feces and blood, which may be the reason why the sensitivity and specificity of SEPT9 and SDC2 methylation are similar in feces and blood (10). This similarity in diagnostic performance highlights the potential of SDC2 as a viable alternative to SEPT9 in CRC screening and diagnosis.

Our study aimed to address the limitations of previous meta-analyses. In 2022, Wang et al. (29) reviewed 12 studies focusing on the diagnostic performance of SDC2 methylation as a potential biomarker for early colorectal cancer screening. Their meta-analysis reported a pooled sensitivity of 0.81 (95% CI 0.74–0.86) and a pooled specificity of 0.95 (95% CI 0.93–0.96), indicating high diagnostic accuracy for SDC2 (29). Our study corroborates these findings, demonstrating similar sensitivity and specificity for SDC2. However, a significant advancement in our study is the direct head-to-head comparison of SDC2 and SEPT9, which was not explored in Wang et al.’s analysis. This comparison in both plasma and fecal samples allows for a more robust evaluation of their diagnostic efficacy.

In comparing our study to another previous meta-analysis conducted by Hariharan et al. (30), several notable advantages emerge. Hariharan et al. (30) included 19 studies focusing exclusively on the diagnostic performance of the SEPT9 methylation test for early colorectal cancer (CRC) detection. They reported a pooled sensitivity of 69% (95% CI: 62–75%) and a specificity of 92% (95% CI: 89–95%), which aligns closely with the findings of our analysis (30). However, a key limitation of their work was the lack of comparison with other methylation sites and biomarkers, as well as the inclusion of relatively older studies. Our study, in contrast, expands upon this foundation by incorporating more recent literature up to October 2024, providing a more updated and comprehensive dataset. This allows for a broader evaluation of the diagnostic utility of both SDC2 and SEPT9 methylation tests, highlighting their respective strengths and weaknesses in CRC screening. One of the major advantages of our study is the comprehensive dual-sample analysis (plasma and fecal), which provides a more holistic view of the diagnostic performance of SDC2 and SEPT9. This head-to-head comparison is pivotal for clinical decision-making, as it enables a more informed choice between the two tests based on sample type availability and patient preferences.

Our study shows that SDC2 and SEPT9 have similar sensitivity and specificity in detecting CRC, whether in plasma or fecal samples. However, these two diagnostic tools each have their advantages in terms of availability and cost-effectiveness. SEPT9 testing has been widely researched and applied, showing high specificity but at a higher cost, while SDC2 testing may offer advantages in terms of cost and operational simplicity. The differing mechanisms and advantages of these diagnostic tools suggest that they might be complementary to some extent. In the future, combining these two in a joint diagnostic model or in conjunction with other site detections may improve overall diagnostic performance. The choice of which diagnostic tool to use in clinical practice should depend on the specific circumstances of the patient, the availability of sample types, and patient preferences.

Some limitations of the current meta-analysis should be considered when interpreting the results. Firstly, the heterogeneity of the included studies may have affected the overall sensitivities or specificities of SDC2 and SEPT9. We therefore tried to identify the sources of heterogeneity by performing meta-regression and sensitivity analysis. The region and study design might be sources of sensitivity heterogeneity for SDC2 and SEPT9, while the number of study participants could be a source of specificity heterogeneity for SDC2. While we identified these factors, we cannot rule out other variables such as preprocessing methods, PCR replication numbers, PCR loading volumes, sampling methods, sample volumes, and storage methods, which may also influence heterogeneity. Future research should aim to investigate these factors more thoroughly and explore their effects within specific subgroups to enhance the robustness of findings. Secondly, in subgroup analysis, the lack of head-to-head comparison studies in our meta-analysis for plasma (six studies) and stool(three studies) samples is a limitation. Therefore, well-designed prospective head-to-head studies focusing on specific sample sources are needed to confirm the findings of this meta-analysis.

## 5 Conclusion

Our meta-analysis indicates that SDC2 demonstrates similar sensitivity and specificity to SEPT9 in the early detection of colorectal cancer. However, the high heterogeneity may impact the evidence of the results, further larger sample prospective research is required to confirm these findings.
