# Supplementary material for: Comparative analysis of SDC2 and SEPT9 methylation tests in the early detection of colorectal cancer: a systematic review and meta-analysis
Source: Front Med (Lausanne). 2024 Dec 10;11:1460233. doi: 10.3389/fmed.2024.1460233 (PMC11666333; doi:10.3389/fmed.2024.1460233)

Supplementary Table 1 Search strategy in PubMed, Embase and Web of Science.

| Database | Search strategy |
| --- | --- |
| PubMed | ("Syndecan-2"[Mesh] OR "syndecan-2"[Title/Abstract] OR "SDC2"[Title/Abstract] OR “SEPT9”[Title/Abstract] OR “Septin-9”[Title/Abstract] OR “mSEPT9”[Title/Abstract]) AND ("Colorectal Neoplasms"[Mesh] OR “Colorectal Tumor”[Title/Abstract] OR “Colorectal Neoplasm”[Title/Abstract] OR “Colorectal Cancer”[Title/Abstract]) |
| Embase | ('syndecan 2'/exp OR ‘syndecan-2’:ab,ti OR ‘SDC2’:ab,ti OR 'sept9 gene'/exp OR 'SEPT9’:ab,ti OR ‘Septin-9’:ab,ti OR ‘mSEPT9’:ab,ti) AND ('colorectal tumor'/exp OR 'Colorectal Tumor':ab,ti OR ‘Colorectal Neoplasm’:ab,ti OR ‘Colorectal Cancer’:ab,ti) |
| Web of Science | (TS=("Syndecan-2"OR "syndecan-2"OR "SDC2" OR “SEPT9” OR “Septin-9” OR “mSEPT9”)) AND TS=("Colorectal Neoplasms" OR “Colorectal Tumor” OR “Colorectal Neoplasm” OR “Colorectal Cancer”) |

Supplementary Figure 1 Leave-one-out sensitivity analysis of SDC2’s sensitivity for colorectal cancer.


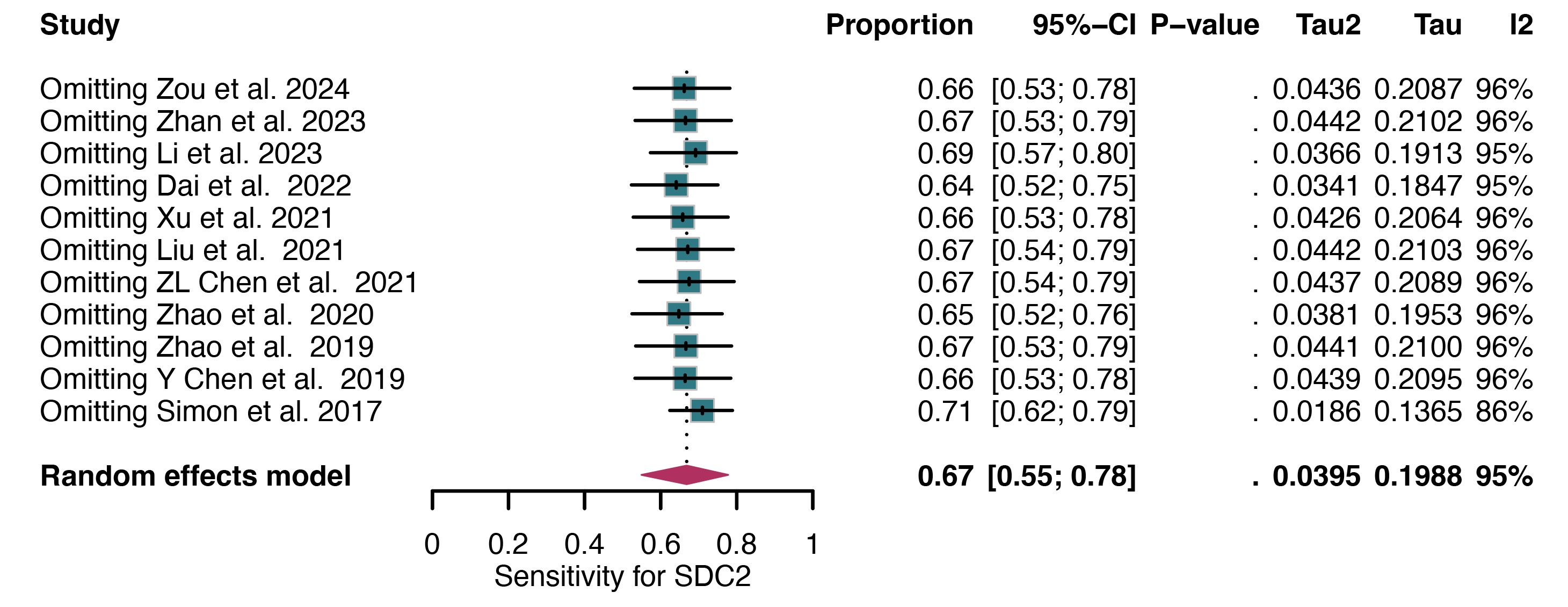


Supplementary Figure 2 Leave-one-out sensitivity analysis of SEPT9’s sensitivity for colorectal cancer.


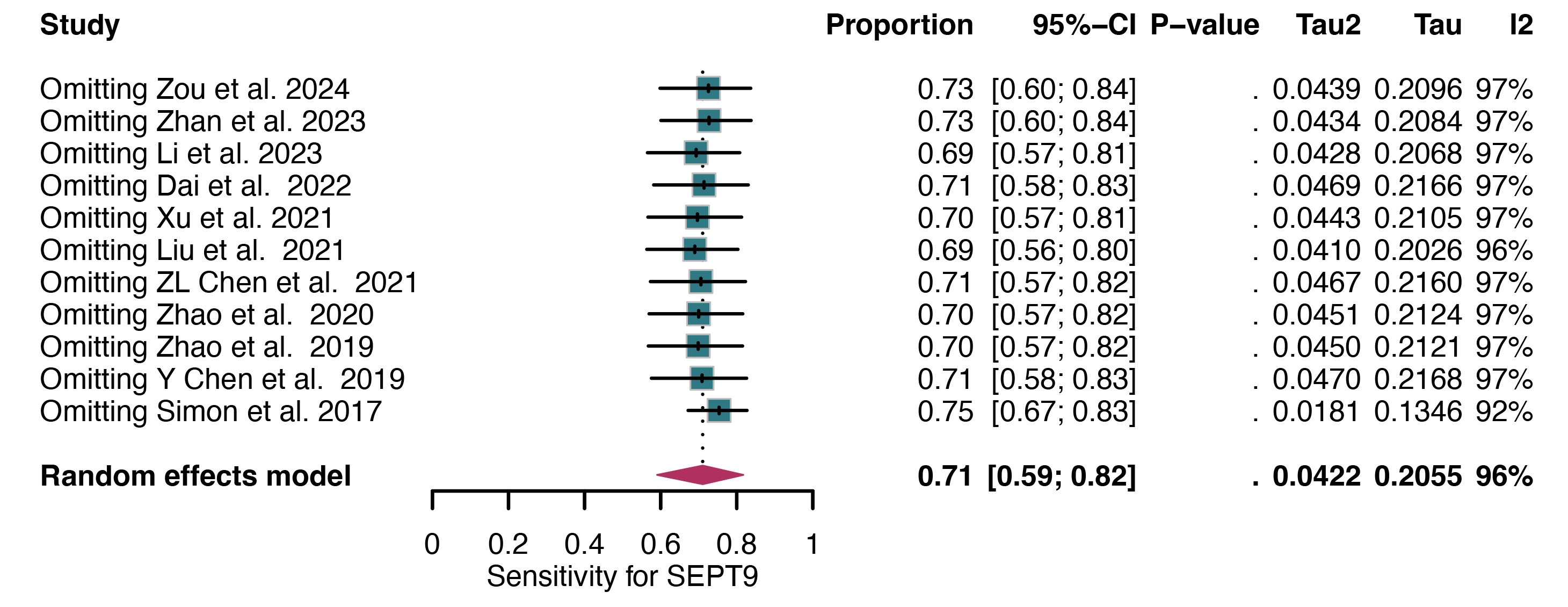


Supplementary Figure 3 Leave-one-out sensitivity analysis of SDC2’s specificity for colorectal cancer.


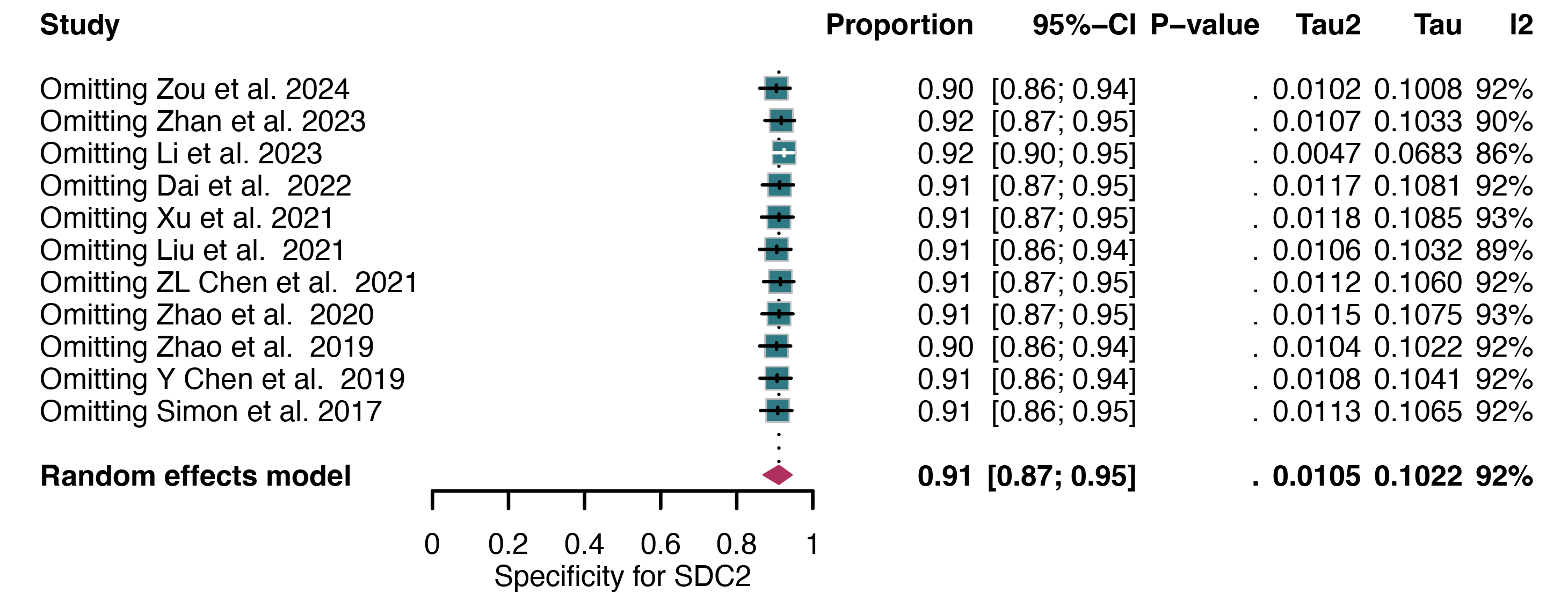


Supplementary Figure 4 Leave-one-out sensitivity analysis of SEPT9’s specificity for colorectal cancer.


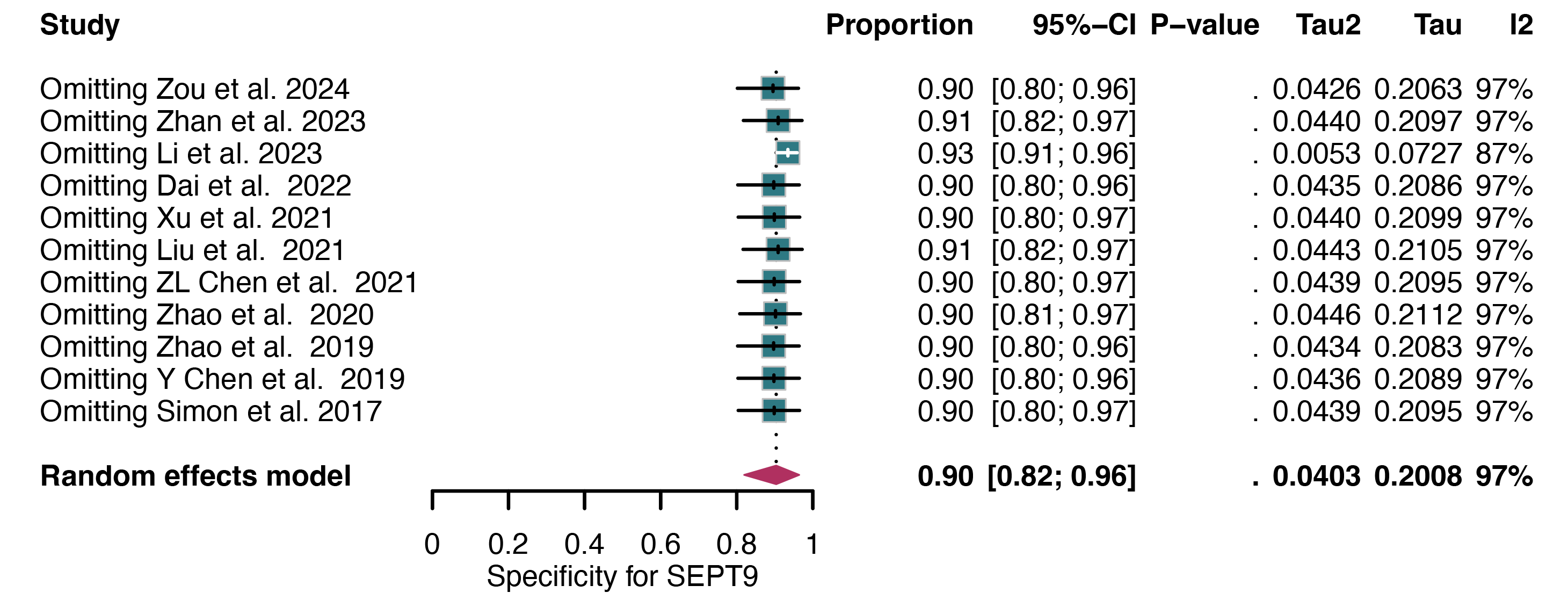


Supplementary Figure 5 Funnel plot illustrating the sensitivity of SDC2 for colorectal cancer.


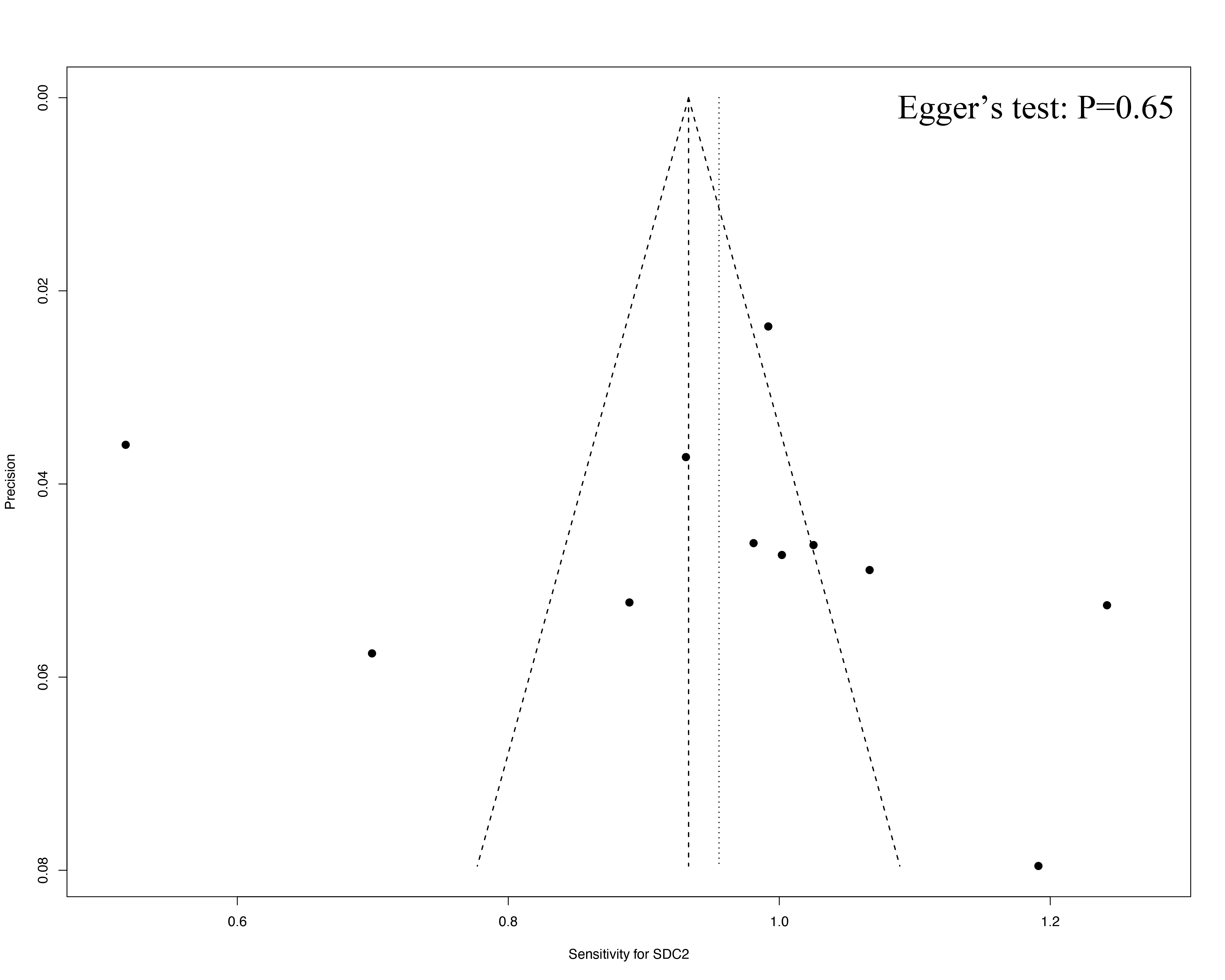


Supplementary Figure 6 Funnel plot illustrating the sensitivity of SEPT9 for colorectal cancer.


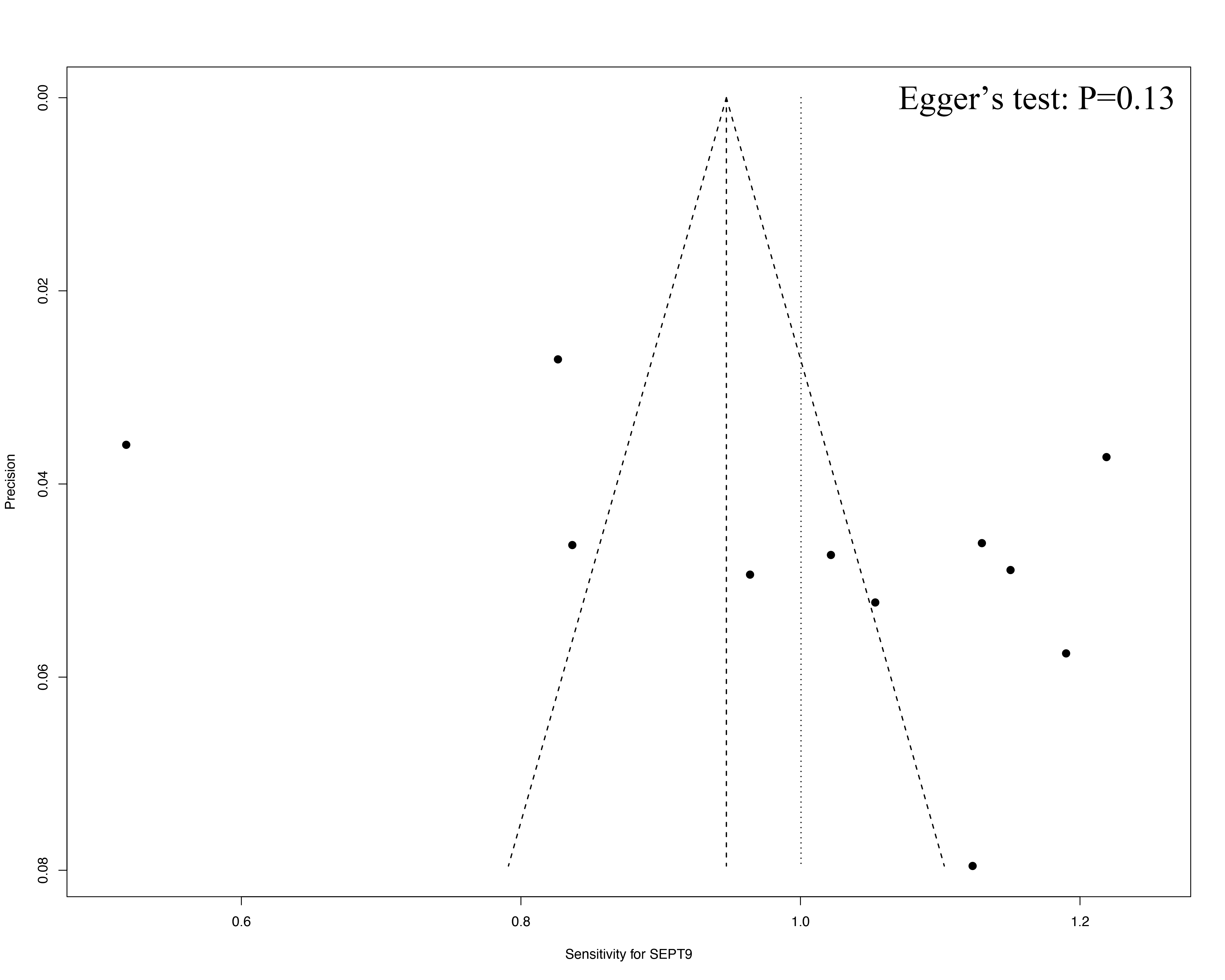


Supplementary Figure 7 Funnel plot illustrating the specificity of SDC2 for colorectal cancer.


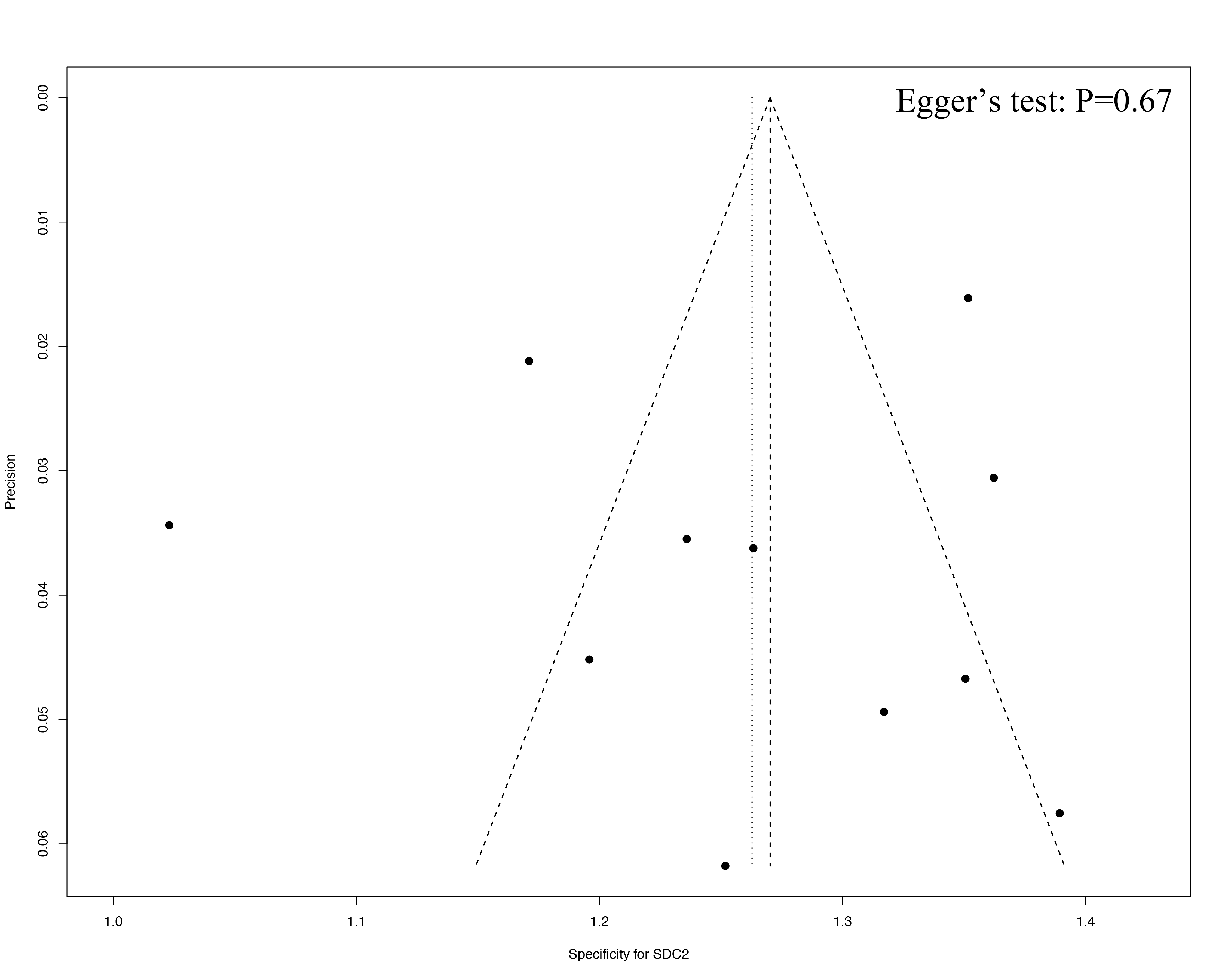


Supplementary Figure 8 Funnel plot illustrating the specificity of SEPT9 for colorectal cancer.


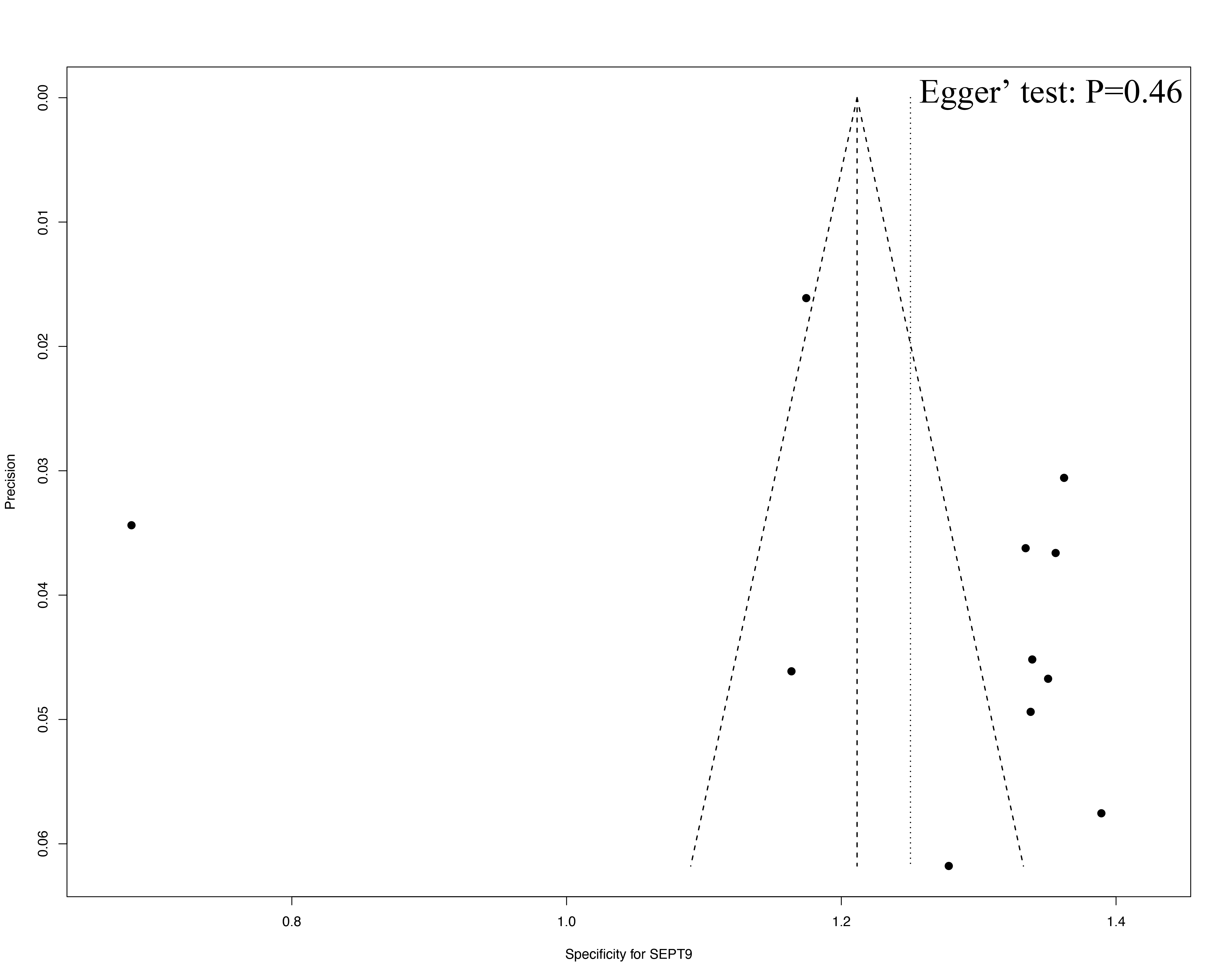

Supplement: Supplementary file 1 [file Data_Sheet_1.docx]
